# The trajectories of CD4 T lymphocytes over time in patients who have defaulted on treatment for tuberculosis in a cohort of people living with HIV, Recife/PE

**DOI:** 10.1371/journal.pone.0299244

**Published:** 2024-03-13

**Authors:** Rossana Cunha, Demócrito de B. M. Filho, Maria de Fátima P. M. Albuquerque, Heloísa R. Lacerda, George T. N. Diniz, Ulisses R. Montarroyos, Laura C. Rodrigues, Líbia Cristina R. Vilela Moura, Ricardo A. A. Ximenes

**Affiliations:** 1 Postgraduate Program in Tropical Medicine, Universidade Federal de Pernambuco, Recife, Brazil; 2 Postgraduate Program in Health Sciences, Universidade de Pernambuco, Recife, Brazil; 3 Aggeu Magalhães Research Center (CPqAM), FIOCRUZ, Recife, Brazil; 4 NEG—Aggeu Magalhães Research Center, Fundação Oswaldo Cruz, Recife, Brazil; 5 Institute of Biological Sciences, Universidade de Pernambuco, Recife, Brazil; 6 Department of Infectious Disease Epidemiology, London School of Hygiene and Tropical Medicine, London, United Kingdom; UFSJ: Universidade Federal de Sao Joao del-Rei, BRAZIL

## Abstract

**Background:**

The CD4 T lymphocyte count in people living with HIV (PLHIV) is a predictor for the progression of the disease (AIDS), survival and response to antiretroviral treatment (ART). A CD4 T lymphocyte count of less than 200 cells/mm3 is indicative of a greater risk for the onset of opportunistic diseases and death. Defaulting on treatment for tuberculosis (TB) may impact immune recovery in PLHIV who are taking ART. The aim of this study was to investigate an association of the CD4 lymphocyte with TB treatment Trajectory and with death.

**Methods:**

A cohort of PLHIV over eighteen years of age and who were taking ART and who had defaulted on pulmonary TB treatment. Latent Class analysis was used to identify different trajectories of CD4 T lymphocyte counts over time.

**Results:**

Latent class 1 (High CD4 trajectory) grouped individuals together who were characterized as maintaining a low probability (0 to 29%) of a CD4 count ≤ 200 cells/mm^3^over time, while latent class 2 (Low CD4 trajectory) grouped individuals together with a high probability (93% to 60%), and latent class 3 (Fluctuating CD4 trajectory), grouped individuals with a fluctuating probability (66% to 0%). The chance of defaulting on treatment earlier (≤ 90 days) was four times higher in latent class 2 (Low CD4 trajectory). Although there was no statistical significance, there was a higher frequency of deaths in this same latent class.

**Conclusion:**

Individuals with a high probability of a CD4 count ≤ 200 cells/ mm^3^ should be monitored in order to avoid treatment default and thereby prevent death. New studies should be conducted with a larger sample size and a longer follow-up time in PLHIV who initiated ART treatment early so as to support clinical decisions for a better understanding of immune behavior.

## Introduction

Tuberculosis (TB) is the leading cause of morbidity and mortality among people living with HIV (PLHIV) [1]. The synergism between the two diseases contributes to an unfavorable outcome both in terms of TB control and in the progression of AIDS.

The CD4 T lymphocyte count is a predictor for the progression of disease (AIDS), survival and response to ART in PLHIV. A CD4 T lymphocyte count of less than 200 cells/mm^3^ is indicative of a greater risk for the onset of opportunistic diseases and death [2–4]. The use of ART reduces the chance of PLHIV developing TB, decreases the risk of HIV transmission, and also has an impact on reducing mortality by up to 50% [2, 5–8].

Some studies have demonstrated the dynamics of CD4 T lymphocytes in PLHIV who were taking ART and had initiated treatment for TB [9–11]. However, neither the trajectory of CD4 T lymphocytes after TB treatment default has been explored, nor whether this trajectory would be a predictor of death.

In a recent PLHIV cohort study, we demonstrated that patients taking ART who defaulted on TB treatment were four times more likely to die when compared to those who did not default on TB treatment [12]. Deaths occurred at different points in time after treatment default (18% of deaths occurred in the 1st year, 33% in the second year, and 48% after two years or more), suggesting that the deaths may have occurred either through the progression of TB, and the impact of longer-term TB on the course of HIV infection [12].

The aim of the present study was to identify different trajectory patterns of CD4 T lymphocyte counts and their association with TB treatment default and death.

## Materials and methods

Individuals over eighteen years of age who had initiated treatment for pulmonary TB and who were taking ART were selected from a cohort of PLHIV in Recife/PE. The individuals were treated at two referral hospitals in the state of Pernambuco: Hospital Correia Picanço (HCP–SES/PE) and Hospital Universitário Oswaldo Cruz (HUOC–UPE). This research contained two components: one retrospective (July/2007 to December/2016) and the other prospective (January/2017 to December/2018). Two groups were observed over time: a group of individuals who had defaulted on TB treatment and another group who had not. The follow-up time was twelve years. In our study TB cases were considered as those diagnosed of pulmonary TB by the attending physician (according to Ministry of Health of Brazil guidelines, that is based on clinical findings, direct investigation of Acid-fast bacillus–AFB–smear for *Mycobacterium tuberculosis)* and notified to the Notifiable Diseases Information System (SINAN/MS). We defined “default on treatment for TB” according to the guidelines of Brazilian Ministry of Health. Default from TB treatment was defined as any patient who failed to attend their pre-book return appointment at the health center for more than 30 consecutive days [13].

There was a total of 12 patients who met the inclusion criteria of this study and of a previous study conducted by our group [12] and therefore were included in the analysis of both papers.

### Latent class analysis

In latent class analyses, unobserved phenomena (latent variables) are investigated using data from observed variables collected by means of observational classifications or continuous values. Within this context, combinations of events may present a level above or below a certain cut-off point for each observed variable in the study, determining the probabilities for each individual to belong to a certain group in constructing the latent variable. This technique allows to group different individual trajectories according to their similarities, and therefore to construct patterns of evolution. Grouping different individual trajectories according to their similarities. It is particularly useful in situations in which there is heterogeneity in the evolution of a characteristic as the CD4 count. A limitation of this technique is that it is not clear the exact effect of sample size on the ability to identify the set of underlying latent classes [14].

In order to analyze the latent classes in the present study, we used the levels of the CD4 T lymphocyte tests which had been performed over time, before, during and after treatment for TB.

Different CD4 T lymphocyte count cut-off points were tested (100, 200, 350, 500 cells/mm3) in order to identify the most appropriate latent class model for analysis and adopted the CD4 cut-off point of ≤ 200 cells/ mm^3^, thereby determining the probabilities for each of the individuals belonging to a certain class. Each TB treatment undertaken by the patient was adopted as a basis, observing the TB treatment default. Thus, the same patient could contribute with the levels of more than one default.

For analytical purposes, the CD4 counts were observed over five periods of time: before initiating treatment for TB (T1 and T2), during the treatment period, corresponding to 180 days (T3), and after treatment (T4 and T5). With the exception of the T3 period, the others were 365 days. For each period (T1/T2/T3/T4/T5) the CD4 T lymphocyte counts were observed, and when there was more than one level, we used the median value of that period in the latent class analysis. The CD4 counts used for the analysis were those obtained up to 730 days (two years) before the initiation date of TB treatment, and those obtained up to 910 days after the initiation date of treatment. We placed individuals with a cut-off point of CD4 ≤200 cells/mm^3^ during each period into 2 groups: default and non- default of TB treatment.

In the analysis, to select the most suitable model, we assessed the model with two and three latent classes, observing the values of the parameters: entropy, AIC, BIC and adjusted BIC, as well as the p-values for the adjustment of each model. We selected the model with three latent classes and identified the patterns of the CD4 T lymphocyte count trajectories (LC1 = High CD4 trajectory; LC2 = Low CD4 trajectory; LC3 = Fluctuating CD4 trajectory).

We performed a complementary analysis to investigate the association between defaulting on TB treatment and the CD4 trajectories identified in the latent class analysis. We also investigated the association between these trajectories and death. We calculated the odds ratio, the “odds ratio OR” and the p-value, using the binomial logistic regression model with a 95% confidence interval. For the latent class analysis we used the R Project for the Statistical Computing program R 4.1.3.

### Ethical considerations

The study was approved by the Ethics Committee of the Federal University of Pernambuco (Registry by SISNEP FR 3.564.145/ CAAE– 15424019.9.0000.5208/REGISTRO/CEP/CCS/UFPE). All the participants of the study provided a written informed consent.

## Results

**Fig 1:**
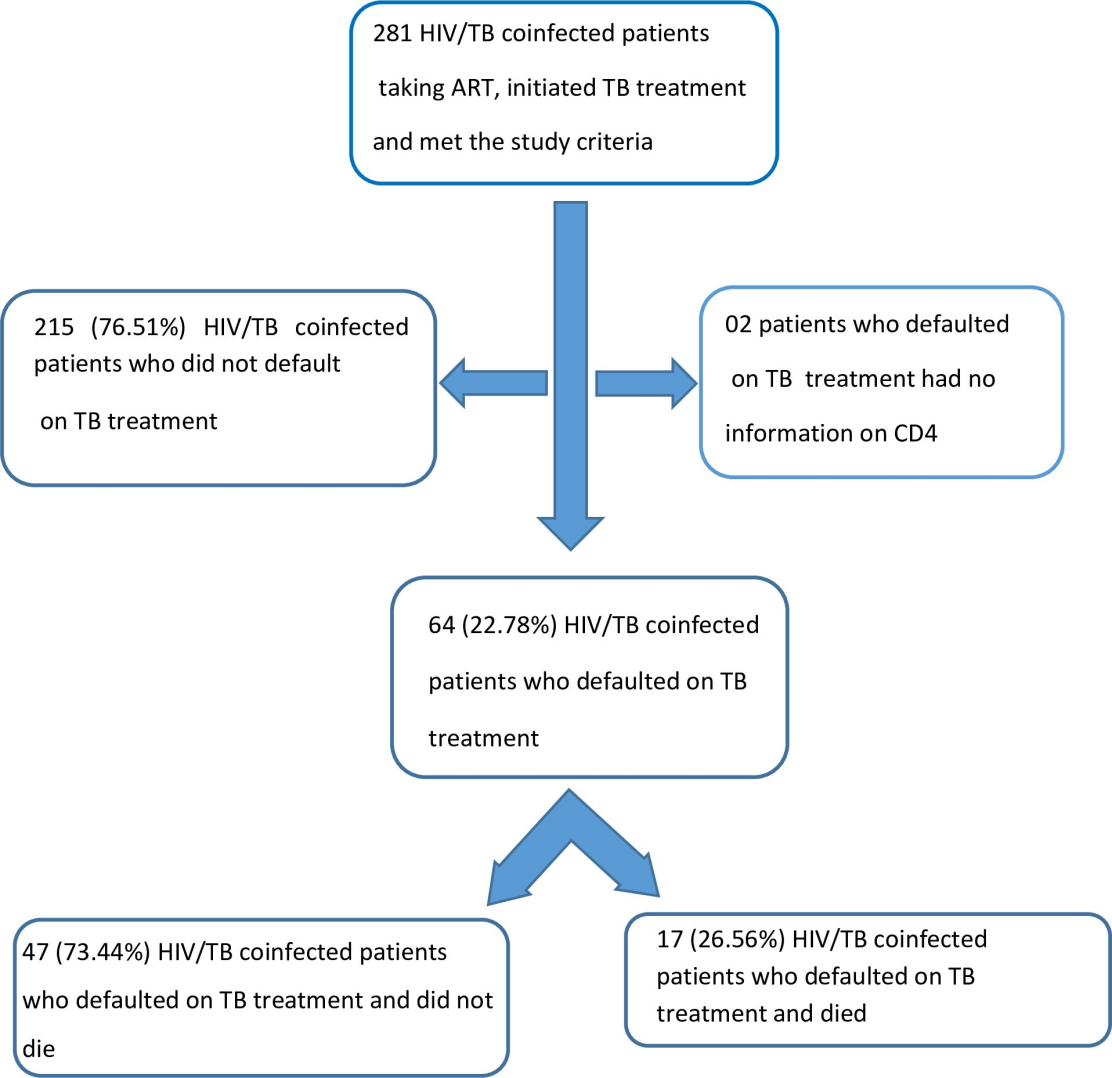
Selection of research participants in a cohort of PLHIV taking ART who initiated TB treatment, Recife/ PE, Brazil.

A total of 281 HIV/TB coinfected patients were taking ART, initiated TB treatment and met the study criteria. Two participants who defaulted TB treatment presented no information on CD4 counts during the study period. A total of 215 (76.51%) participants did not default on TB treatment and 64 (22.78%) defaulted on TB treatment. Of the 64 patients coinfected with HIV/TB who defaulted on TB treatment, 17 (26.56%) died (Fig 1).

Most of the participants were male, with a total of 206 (73.31%). From a total of 281 individuals, 215 (76.51%) did not default on TB treatment and 64 (22.78%) defaulted on treatment (Table 1).

**Table 1:** Demographic and clinical characteristics of participants in a cohort of PLHIV taking ART who initiated TB treatment, Recife/ PE, Brazil.

Variable	n (%)
**Sex**	
Male	206 (73.31%)
Female	75 (26.69%)
**Age**	
< 30 years	43 (15.30%)
30 to 49 years	188 (66.90%)
≥ 50 years	50 (17.80%)
**TB treatment default**	
No default	215(76.51%)
Default	64(22.78%)
Defaulted on TB treatment but had no information on CD4	02(0.71%)

Of the 64 participants who defaulted on TB treatment, seven (10.93%) had defaulted twice and 17 (26.56%) died (Table 2).

**Table 2:** Participants of a cohort of PLHIV taking ART who defaulted on TB treatment, Recife/PE, Brazil.

Characteristics	n (%)
N° of TB treatment defaults	71
N° of individuals who defaulted on TB treatment	64
N° of individuals who defaulted twice	7(10.93%)
N° of deaths of individuals who defaulted on TB treatment	17(26.56%)
Time of monitoring the cohort	12 years

Table 3 shows the results of the statistical tests and other parameters used to construct the latent classes and to assess model fit (Table 3).

**Table 3:** Selection of latent classes model in a cohort of PLHIV taking ART who defaulted on TB treatment, Recife/ PE, Brazil.

Evolution of the Model			
**Entropy**	0.8350		
**AIC**	237.1		
**BIC**	275.6		
**Adjusted BIC**	222.0		
**Test statistics**	p-value		
VLMR LR test	**0.0369**		
ALMR LR test	**0.0300**		
BLRT	**0.0300**		
**Assessment of the Classes and of the Model**	**Latent Classes**		
	**CD4 Trajectories**		
	**1**	**2**	**3**
**No. of Observations (Total TB Treatment Defaults)**	**38**	**26**	**7**
**Probabilities (Unconditional)**	0.5352	0.3662	0.0986
**ALC Probabilities (Diagonal)**	0.9410	0.8780	1.0000
**Conditional Probability of CD4 > 200 cells**	**CD4 Trajectories**			
	**1**	**2**	**3**
**T1—Before (730 / 365 days)**			
> 200	**1.000**	**0.067**	**0.385**
≤ 200	0.000	0.933	0.615
**T2—Before (365 / 0 days)**			
> 200	**0.835**	**0.000**	**1.000**
≤ 200	0.165	1.000	0.000
**T3 –Initiating TB Treatment (0 / 180 days)**			
> 200	**0.706**	**0.000**	**0.613**
≤ 200	0.294	1.000	0.387
**T4—Post (180 / 545 days)**			
> 200	**1.000**	**0.000**	**0.159**
≤ 200	0.000	1.000	0.841
**T5—Post (545 / 910 days)**			
> 200	**1.000**	**0.394**	**0.000**
≤ 200	0.000	0.606	1.000

Latent class 1 groups individuals together whose immunological behavior was characterized by a low probability (0 to 29%) of maintaining a CD4 T lymphocyte count ≤ 200 cells/mm^3^ over time. This trajectory is referred to as a High CD4 trajectory.

Latent class 2 groups individuals together whose immunological behavior was characterized by a high probability (93% to 60%) of maintaining a CD4 T lymphocyte count ≤ 200 cells/mm^3^ over time. This trajectory is referred to as a Low CD4 trajectory.

**Fig 2:**
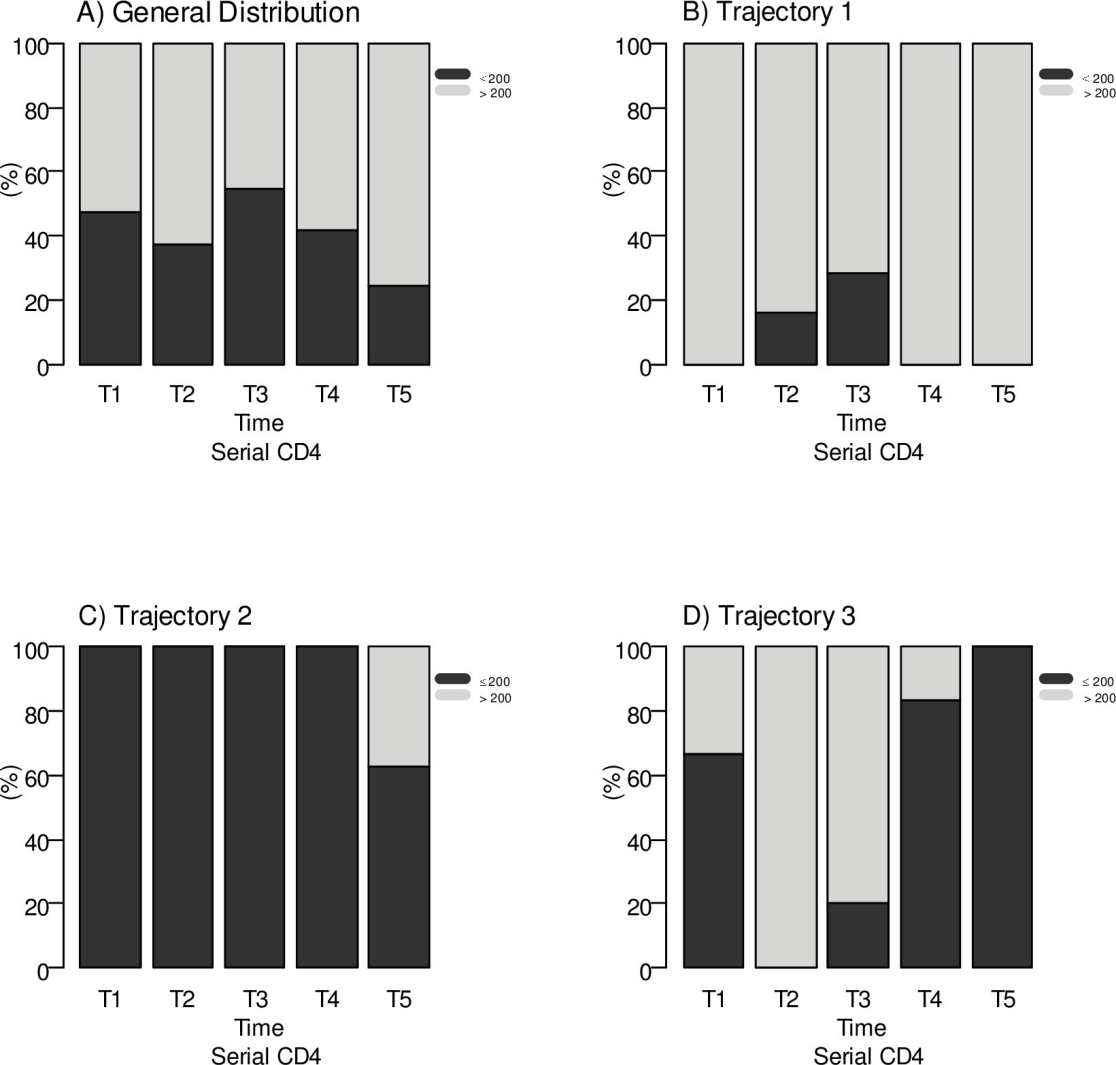
CD4 trajectories of PLHIV in a cohort taking ART who defaulted on TB treatment, Recife/PE, Brazil.

Latent class 3 groups individuals together whose immunological behavior varied over time, characterized by an initial period in which the probability of maintaining CD4 T lymphocytes ≤ 200 cells/mm^3^ decreased until reaching 0%, followed by another moment in which the probability of maintaining CD4 T lymphocytes ≤ 200 cells/mm^3^ increased until reaching 66%. This trajectory is defined as a Fluctuating CD4 trajectory (Fig 2).

The mean CD4, CD8 T lymphocyte count and CD4/CD8 at the start of tuberculosis treatment in HIV patients who initiated tuberculosis therapy were in the High CD4 trajectory were, respectively, 433.74 cells/mm^3^, 1179.14 cells/mm^3^, 0.42. For the patients in the Low CD4 trajectory these values were: CD4 90.36 cells/mm^3^, CD8 654.56 cells/mm^3^, CD4/CD8 ratio 0.13 and for the patients in the Fluctuating CD4 Trajectory: CD4 179.75 cells/mm^3^, CD8 1451 cells/mm^3^, CD4/CD8 ratio 0.1. The mean viral loud for the High CD4 trajectory, Low CD4 trajectory and Fluctuating CD4 trajectory were, respectively, 55115.89 copies/ml, 174441.52 copies/ml, 1678.5 copies/ml.

The observed defaults in the Low CD4 trajectory were four times more likely to occur in the earlier stages of TB treatment. Of the total default with a time of ≤ 90 days, approximately, 50% (21) belonged to the Low CD4 Trajectory, while 18% of those who abandoned treatment after 90 days of the start of TB treatment belonged to the same group (Table 4).

**Table 4:** Association of the CD4 trajectories over time with the time of defaulting on TB treatment, in a cohort of PVHIV, Recife/PE, Brazil.

CD4 Trajectory	Time of Default (days)				CI 95%			
	≤ 90		> 90		OR			p-value
	N	%	N	%		Lower	Upper	
High CD4 Trajectory	19	44.19	19	67.86	1.00			
Low CD4 Trajectory	21	48.84	5	17.86	4.20	1.31	1.35	**0.0157**
Fluctuating CD4 Trajectory	3	6.98	4	14.29	0.75	0.15	3.81	0.7289

There was no statistical significance in the association of deaths with the CD4 trajectories. However, it should be emphasized that among those who died, 52% belonged to the Low CD4 trajectory, while of those who did not die, 34% belonged to this same trajectory (Table 5).

**Table 5:** Association of CD4 trajectories over time with death, in individuals who defaulted on TB treatment, in a cohort of PVHIV, Recife/PE, Brazil.

	Death				CI 95%			
CD4 Trajectories	Yes			No	OR			p-value
	N	%	N	%		Lower	Upper	
High CD4 Trajectory	7	41.18	28	59.57	1.00			
Low CD4 Trajectory	9	52.94	16	34.04	2.25	0.71	7.44	0.1718
Fluctuating CD4 Trajectory	1	5.88	3	6.38	1.33	0.06	12.40	0.8150

**Fig 3:**
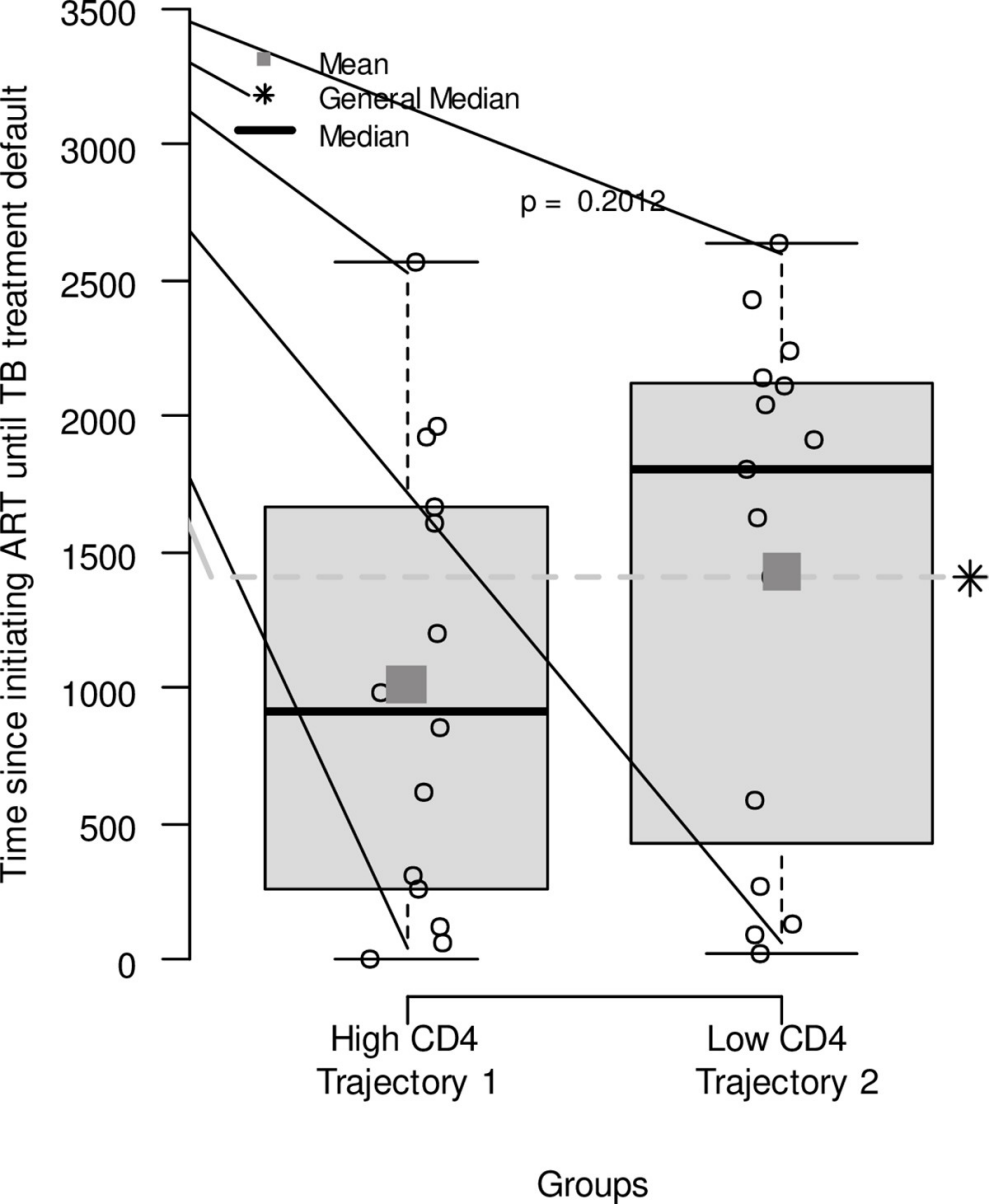
Time since initiating ART until the defaulting on TB treatment according to the CD4 Trajectory in a cohort of PLHIV, Recife/PE, Brazil.

In the High CD4 trajectory (Trajectory 1), the mean time of initiating ART was 1010.79 and the median was 918.50, with the 1st Quartile of 273.00 and the 3rd Quartile of 1655.50. The Low CD4 trajectory (Trajectory 2) presented values of 1430.73 and 1810.00 for the mean and median respectively, with values for the 1st quartile of 428.00 and the 3rd quartile of 2127.00. The difference between the medians was not statistically significant (p-value 0.2012) (Fig 3).

## Discussion

The present study has demonstrated different patterns of CD4 trajectories over time. An association was observed between the Low CD4 trajectory and the time of defaulting on TB treatment, whereby the chance of an earlier default was four times greater in the Low CD4 trajectory. The data have suggested a higher mortality rate in the Low CD4 trajectory although the difference was not statistically significant.

Two other patterns were also observed, one that presented a Low probability of a CD4 count ≤ 200 cells/mm^3^ over time (High CD4 trajectory) and another that presented a greater fluctuation in the probability of a CD4 count ≤200 cells/mm^3^ (Fluctuating CD4 trajectory).

Initiating TB treatment in PLHIV can have a positive impact in terms of immune system recovery, although this potential impact may be compromised by treatment default. The High CD4 trajectory demonstrated an improvement in the immune system one year after initiating and defaulting on TB treatment, revealing an earlier immune recovery, while, in the Low CD4 trajectory, there was a late immune recovery, two years after initiating and defaulting on TB treatment. HIV/TB coinfected individuals taking ART may present a poor immune recovery when compared to monoinfected individuals [11, 15, 16]. This poor recovery may have occurred due to TB infection in PLHIV even when taking ART, depending on the duration of taking ART and the action caused by the HIV virus infection in these individuals. There are some mechanisms that explain this phenomenon, one of which is the Niche concept when there is collagen deposition in the lymphoid tissue that reduces the production of IL7 (Interleukin 7) responsible for activating T lymphocytes, thereby decreasing the production of naive T cells, and limiting immune restoration [17].

Another mechanism would be cell apoptosis, which occurs due to the depletion of CD4 T lymphocytes, brought about by the cytotoxic effect of the viral infection, causing cell death [17, 18]. In these individuals in the High and Low CD4 trajectory, it is possible that these mechanisms were acting with a different intensity, implying different levels of immune compromise and different recovery capacities. Thus, individuals in the Low CD4 trajectory, due to a longer period taking ART, greater impairment of the immune system and for a longer period of time, presented a later, less intense degree of immune recovery.

Around 50% of the individuals who defaulted earlier on TB treatment were in the Low CD4 trajectory and the chance of default occurring earlier was four times greater in this trajectory when compared to the High CD4 trajectory. Although some studies have addressed the CD4 dynamics in HIV/TB co-infected patients, they have not explored the impact of treatment default on this dynamic [9, 10, 19]. The behavior of the immune systemis associated with the onset of TB and the evolution of the disease, and it would be plausible to assume that defaulting on TB treatment interferes with the immune system and that this effect may vary according to the period in which the default occurred, whether earlier or later.

Defaulting on TB treatment is inserted into a multifactorial context that depends on factors related to the disease, the individual, the treatment, the social condition and the health service [20] and, as with ART default, induces the immune failure in HIV/TB co- infected patients [21]. Some studies have indicated risk factors for defaulting on TB treatment in monoinfected or co-infected patients similar to risk factors for ART default: **life habits**: **use of illicit drugs** [22]; **alcoholism** [20, 23–26]; **smoking** [20, 24, 27–29]; **adverse drug reactions** [20, 22]; the **male sex** [28, 30–32]; **HIV coinfection** [33–35]. It is possible that the individuals who do not adhere to HIV treatment are the same as those who do not adhere to TB treatment, leading to a greater impairment of the immune system, as observed in individuals who make up the Low CD4 trajectory.

Our results did not show statistical significance in the association of CD4 trajectories with death in individuals who have defaulted on TB treatment. However, it should be noted that the highest of deaths occurred in the Low CD4 trajectory and that the wide confidence interval suggests that there may have been a problem of power of the study, i.e. the sample size was too small to detect the association. It is well documented that patients with a greater impairment of the immune system are at greater risk of death [36–38], just as defaulting on TB treatment is a predictor of death [12]. Ku et al (2013) suggested that the effect of TB on PLHIV reduces the chance of immune recovery after initiating ART and that this may be due to the biphasic response of the HIV virus. The initial biphasic response is related to an increase of CD4 and their redistribution from tissues to the blood; the second phase of the biphasic response—occurs when there is a true increase in the number of cells [39]. Ku et al (2013) suggest TB in HIV-infected patients could affect the redistribution of lymphocytes. Another explanation could be the inflammatory action of the lymphoid tissue that occurs when there is depletion of CD4 T lymphocytes, hindering immune recovery through propulsive mechanisms for the production of new naive T cells and hemostasis of the CD4 T lymphocytes. The fibrotic damage does not reverse after 6 months of taking ART [18]. This effect may be potentiated by treatment default. Therefore, patients who are undergoing concomitant treatment for both diseases (HIV/TB) and who default on TB treatment may suffer a more severe impairment of the immune system. Consequently, they have a lower chance of immune recovery and a greater risk of death.

This study presents advantages and limitations. The main limitation is the relatively small number of PLHIV who defaulted on TB treatment. Another limitation was the use of the CD4 count over time alone as it does not capture all the complexities of immune recovery. Although it has been used in the literature to assess the immunological response in co-infected patients [40, 41], Wolday *et al*. (2020), studying the Immune recovery in HIV-1 infected patients under long term antiretroviral therapy, found different patterns in the increase of CD4 T cells and CD8 T cells over time and pointed that monitoring CD4/CD8 ratio levels may be as a better biomarker risk for disease progression [42]. On the other hand, this study uses a methodology—latent class analysis—which enables individuals and CD4 evolution over time to be placed into groups with similar characteristics, identifying behavior patterns in CD4 dynamics.

## Conclusion

Individuals with a high probability of CD4 ≤200 cells/mm^3^ should be monitored more closely in order to avoid treatment default and prevent death. New studies should be conducted with a larger sample size and a longer period of follow-up in PLHIV who initiated early treatment of ART in order to support clinical decisions for a better understanding of immune behavior.
